# Supplementary material for: Carnosol, a Rosemary Ingredient Discovered in a Screen for Inhibitors of SARM1-NAD+ Cleavage Activity, Ameliorates Symptoms of Peripheral Neuropathy
Source: Antioxidants (Basel). 2025 Jun 30;14(7):808. doi: 10.3390/antiox14070808 (PMC12291994; doi:10.3390/antiox14070808)

Supporting Information for

Carnosol, a rosemary ingredient discovered in a screen for inhibitors of SARM1-NAD$^+$ cleavage activity, ameliorates symptoms of peripheral neuropathy

Hitoshi Murata*, Kazuki Ogawa, Yu Yasui, Toshiki Ochi, Nahoko Tomonobu, Ken-ichi Yamamoto, Rie Kinoshita, Yoji Wada, Hiromichi Nakamura, Masahiro Nishibori, Masakiyo Sakaguchi

Correspondence should be addressed to Hitoshi Murata: murata@md.okayama-u.ac.jp

This file includes:

Fig. S1, Fig. S2, and full unedited gel for WB figures

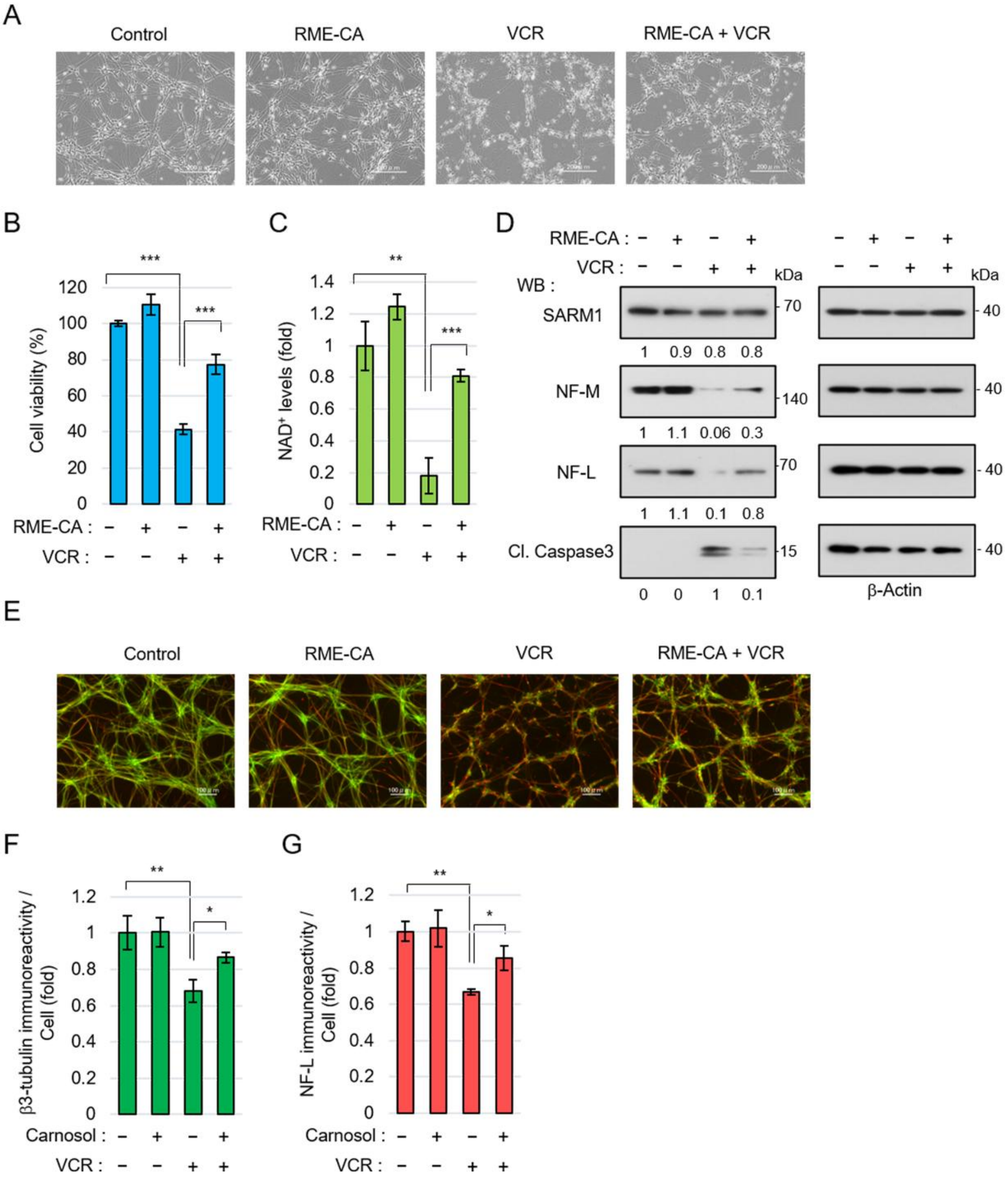

Fig. S1. The rosemary extract RME-CA suppressed VCR-induced neurite degeneration and cell death. (A-G) Human iPSC-derived neurons were treated with 50 nM VCR, and 10 μg/ml RME-CA for 24 h (A-D) or 8 h (E-G). Panels A–D show that (A) cell morphologies, Scale bar: 200 μm, (B) cell viability, (C) NAD^+ levels, and (D) western blotting results of cell lysates at 24 h after the treatment. The band intensities of the indicated proteins were normalized to their corresponding bands of β-Actin in the right

panel, and the values were indicated below each panel. (E) Representative images of neuronal axons at 8 h of treatment. Stain: anti-β3-tubulin and NF-L. Scale bar: 100 μm. (F and G) Immunoreactivity of β3-tubulin and NF-L. The fluorescence intensities of β3-tubulin and NF-L were normalized against nuclear numbers. *p<0.05, **p<0.01, ***p<0.001.

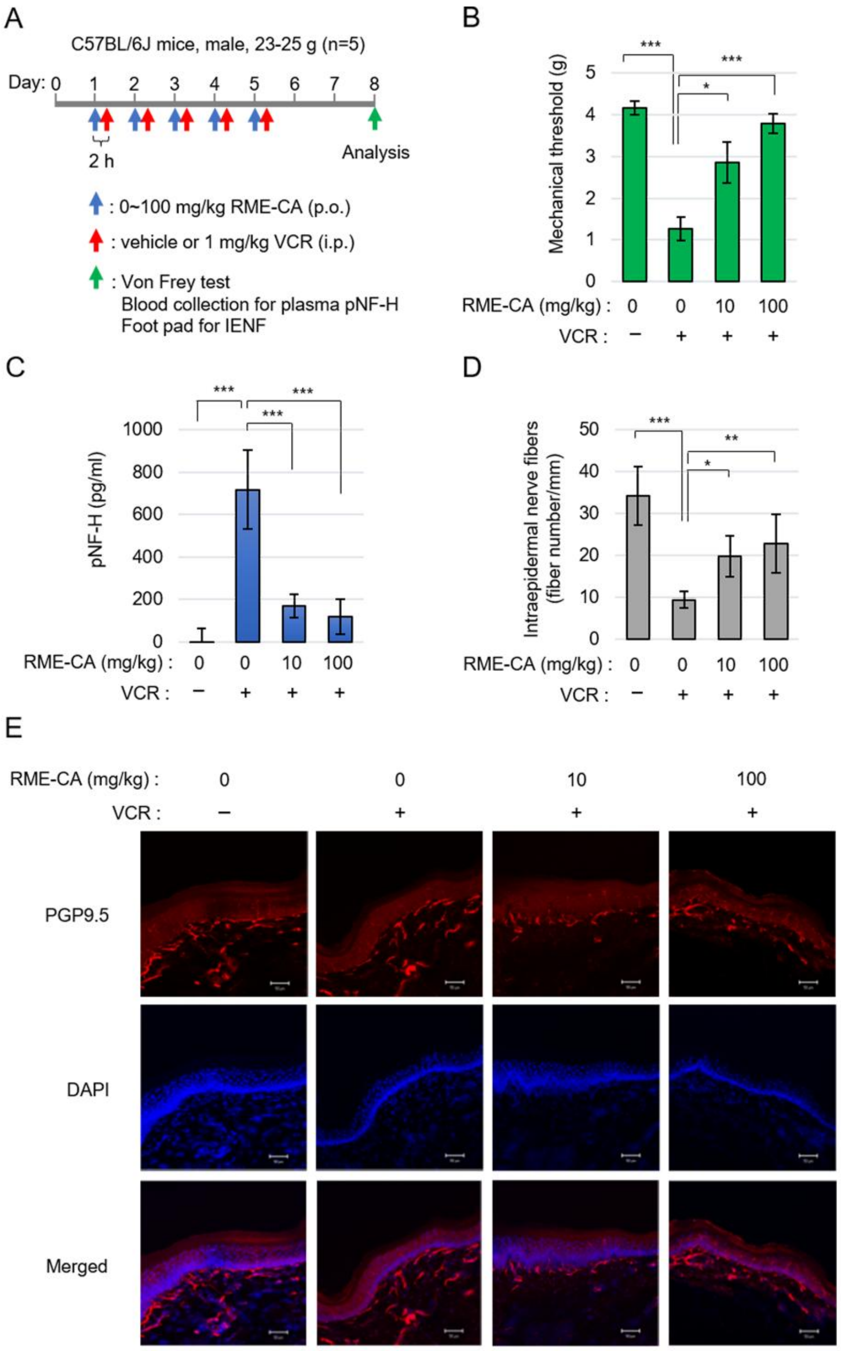

Fig. S2. The rosemary extract RME-CA treatment attenuated VCR-induced peripheral neuropathy. (A) Schematic diagram of the treatment. (B) Quantification of mechanical sensitivity. (C) Quantification of plasma pNF-H levels. (D and E) Changes in INEF density in mice given the treatment. Quantification data (D) and the representative figures

(E). Stain: anti-PGP9.5 and DAPI. Scale bar: 50 μm. $^{*}p<0.05$, $^{**}p<0.01$, $^{***}p<0.001$.

Full unedited gel for Figure 1B

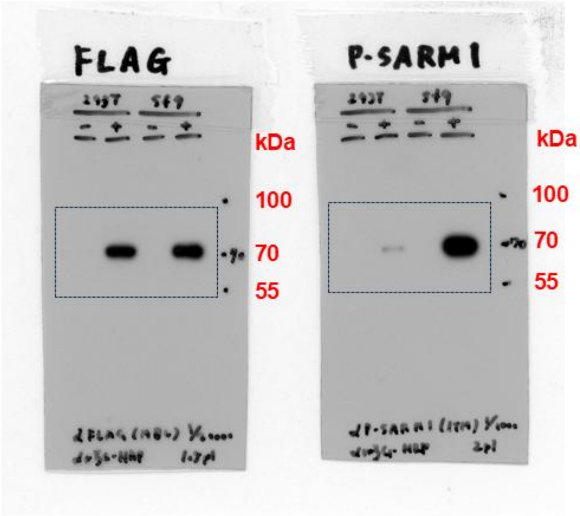

Full unedited gel for Figure 3B

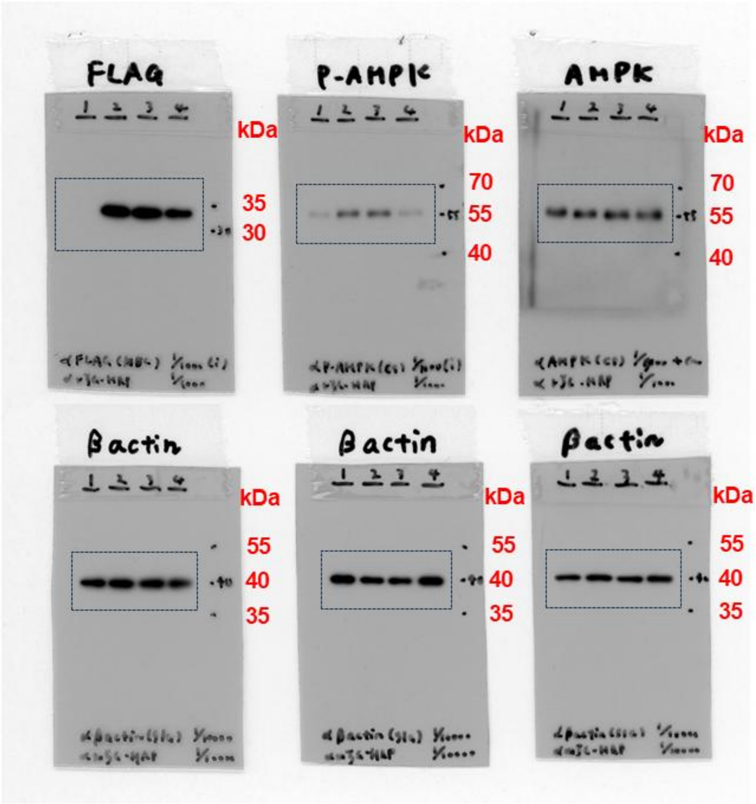

Full unedited gel for Figure 4D

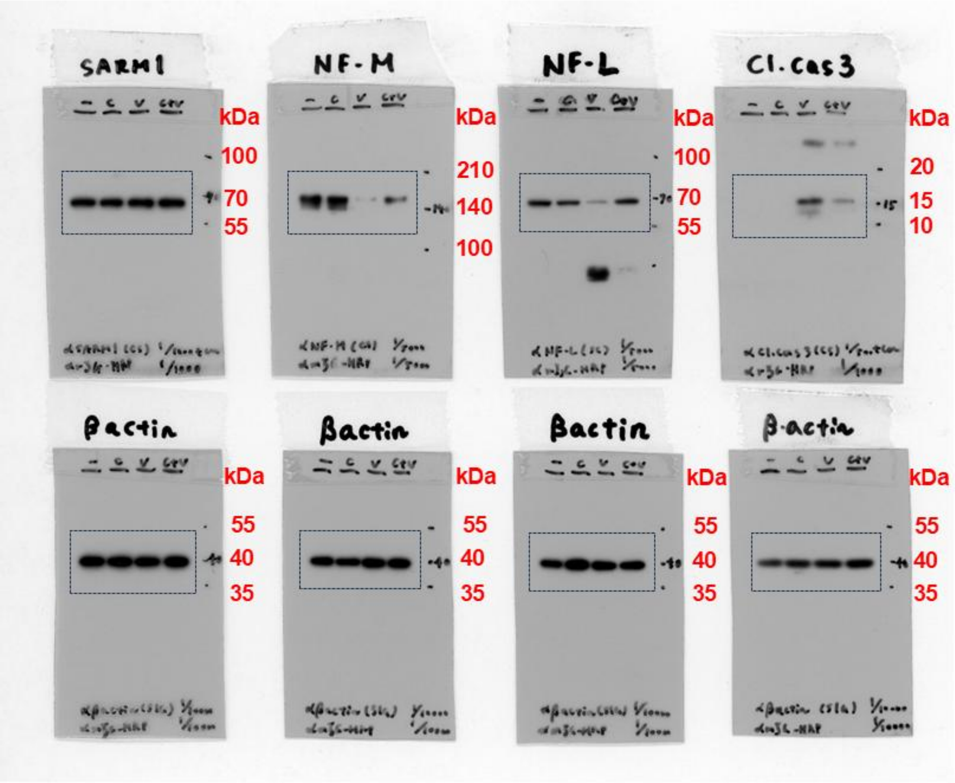

Supplement: Supplementary file 1 [file antioxidants-14-00808-s001.zip › antioxidants-3685756-supplementary.pdf]
